# Comparative review: clinical and pathological heterogeneity in knee *versus* temporomandibular joint osteoarthritis

**DOI:** 10.3389/fbioe.2025.1684481

**Published:** 2025-09-10

**Authors:** Jiannan Zhao, Ruoyi Wang, Songsong Zhu, Zhen Li, Rong Ren, Nan Jiang

**Affiliations:** ^1^ State Key Laboratory of Oral Diseases and National Center for Stomatology and National Clinical Research Center for Oral Diseases, West China Hospital of Stomatology, Sichuan University, Chengdu, Sichuan, China; ^2^ AO Research Institute Davos, Davos, Switzerland

**Keywords:** osteoarthritis, temporomandibular joint, knee, comparison, craniomaxillofacial surgery

## Abstract

Temporomandibular joint osteoarthritis (TMJOA) remains a clinically underrecognized and insufficiently studied disorder, despite exerting a comparable impact on quality of life as knee osteoarthritis (knee OA). TMJOA can lead to chronic pain, limited mouth opening, joint dysfunction, and craniofacial deformities, yet it receives disproportionately less research attention and lacks standardized diagnostic and therapeutic frameworks. While TMJOA and knee OA share several hallmark pathological features—including cartilage degeneration, subchondral bone remodeling, and synovitis—these manifestations are shaped by joint-specific anatomical and biomechanical environments, resulting in distinct disease trajectories. Current evidence highlights that the pathogenesis of TMJOA remains poorly defined, with unresolved questions surrounding the role of mechanical loading in altering the cartilage microenvironment, the mechanisms underlying pathological calcification, and the influence of sex hormones such as estrogen and progesterone on disease onset and progression. In contrast, decades of knee OA research have yielded validated preclinical models, detailed molecular insights, and emerging regenerative strategies. This review systematically compares the two forms of osteoarthritis from clinical, anatomical, and pathological perspectives. We propose that TMJOA research may benefit significantly from cross-joint insights derived from the more extensively studied knee OA. Cross-comparative approaches not only provide a valuable framework for understanding joint-specific disease mechanisms but also offer new directions for the development of targeted therapies and diagnostic tools tailored to TMJOA. Bridging the current knowledge gap through interdisciplinary and translational research may ultimately improve outcomes for patients affected by this overlooked joint disease.

## 1 Introduction

The knee joint and temporomandibular joints (TMJ) are among the most frequently used joints in the human body. The knee is essential for locomotion, while the TMJ, one of the most complex joints, is critical for chewing, speaking, and breathing. Osteoarthritis (OA) is a common degenerative joint disease marked by cartilage degradation, subchondral bone remodeling, and synovial inflammation, leading to pain, stiffness, and functional impairment.

Knee OA is highly prevalent, affecting approximately 14.6% of the population, with incidence increasing with age. It is a major cause of disability worldwide and has been extensively studied in terms of epidemiology and pathogenesis (Cross et al., 2014). By contrast, TMJOA is similarly prevalent (8%–16%) and equally detrimental to patients’ quality of life, causing pain, restricted mouth opening, slowly progressive craniofacial deformities, and even ankylosis, yet it has received disproportionately limited research attention (Wang et al., 2025).

Marked disparities exist between knee OA and TMJOA in terms of basic research, clinical studies, therapeutic development, and funding. For instance, the annual number of total knee replacements is nearly 2,000 times that of TMJ replacements (Bielajew et al., 2021). While research in knee OA has led to detailed insights into inflammatory pathways, cartilage degradation, and therapeutic strategies, TMJOA lacks well-established diagnostic and treatment frameworks.

Given anatomical and pathological similarities between the two conditions, knee OA research offer valuable reference points for advancing TMJOA studies (Table 1). This review aims to compare knee OA and TMJOA from clinical and basic science perspectives, highlighting how cross-joint insights may promote mechanistic understanding and therapeutic innovation for TMJOA.

**TABLE 1:** Overview of comparison of clinical symptoms to pathology in knee OA and TMJOA.

Differential item	Knee OA	TMJOA	Differences	References
Prevalence	∼14.6%	8%–16%	similar	Cross et al. (2014), Kalladka et al. (2014)
PubMed articles^a^	2167 (in 2024)	404 (in 2024)	Knee OA articles far outnumber TMJOA articles	-
Symptoms	Persistent pain, joint stiffness, functional limitation	Joint clicking, limited mouth opening, occlusal pain	Both present with pain and restricted joint function	Glyn-Jones et al. (2015), Ahmad et al. (2009)
Pathological Features-cartilage degeneration	Severe surface fissures, extensive fibrosis	Fissures predominantly in the deep layer, milder surface damage	Knee OA shows cartilage surface fissures, absent in TMJOA	Li et al. (2021), Wallace et al. (2009)
Pathological Features-subchondral bone	Sclerosis, cysts and osteophyte	Erosion, osteophyte, flattening, and pseudocyst-like lesions	Subchondral bone remodeling in TMJOA greatly affects condylar morphology	(Link et al., 2003); (Embree et al., 2011)
Pathological Feature-pathological calcification	Double tidemark phenomenon with calcification at top and bottom layers	Upward shift of the calcification front, absence of double tidemark phenomenon	Pathological calcification in knee OA is characterized by a “double tidemark,” absent in TMJOA	Hawellek et al. (2016), Wang et al. (2022b)
Pathological Features-Synovitis	Significant synovial thickening and inflammatory response	Synovial thickening with increased vascularization	Similar, both are OA-promoting factors	Scanzello and Goldring (2012), Benito et al. (2005)

^a^ PubMed was searched using the following keyword schemes: ([([tibiofemoral] OR [knee]) AND ([cartilage] OR [meniscus])]) and ([([temporomandibular] OR [jaw]) AND ([cartilage] OR [meniscus] OR [disc] OR [disk])]).

## 2 Clinical heterogeneity

### 2.1 Anatomy and histology

The knee and TMJ both are synovial hinge joints with articular surfaces and fibrocartilaginous structures (the meniscus in the knee and the articular disc in the TMJ) that contribute to joint stability and function. The temporomandibular joint is capable of both rotational and translational movements, while the knee joint, divided into the tibiofemoral and patellofemoral joints, enables flexion, extension, and rotational movements.

Biomechanically, the forces exerted on the knee joint during light jogging can exceed four times body weight (approximately 3,080-3,600 N) (D’Lima et al., 2012). In contrast, occlusal loading on the TMJ approximates body weight (770–900 N) (Pizolato et al., 2007). The main types of loads applied in both joints are compressive and shear loads, but tensile loads play a greater role in the TMJ than in the knee (Patel et al., 2019). Notably, occlusal force is modulated by peripheral and central neuromuscular feedback, maintaining TMJ loading within physiological limits (Ogino and Tadi, 2023).

Structurally, TMJ condylar cartilage consists of fibrocartilage, containing both type I and type II collagen, unlike the hyaline cartilage in the knee (Wadhwa and Kapila, 2008). TMJ cartilage is classified as secondary cartilage, forming in conjunction with intramembranous ossification, whereas knee cartilage originates as primary cartilage through endochondral ossification (Breeland et al., 2023). TMJ cartilage also exhibits enhanced regenerative capacity and greater potential for interstitial growth.

Subchondral bone in both joints includes the subchondral plate and trabecular bone. While structurally similar, subchondral bone dynamically adapts to mechanical loading by remodeling trabecular orientation and density in response to stress (Goldring and Goldring, 2016). Continuous bone modeling adjusts the contour and geometry of the subchondral region to maintain joint function under varying load conditions.

### 2.2 Epidemiology

Knee OA is the most prevalent form of OA, affecting approximately 365 million people worldwide (H et al., 2022). Among individuals aged 60 and above, symptomatic knee OA occurs in about 10% of men and 13% of women, with an overall prevalence of 14.6% (Zhang and Jordan, 2010). The prevalence is expected to rise with population aging and increasing rates of overweight and obesity. A cross-sectional study in Greece found that knee OA was more common in women, increased with age, and was more prevalent in rural areas. Additionally, obesity and low educational attainment were identified as risk factors (Andrianakos et al., 2006).

In contrast, epidemiological data on TMJOA are limited and inconsistent due to variations in diagnostic criteria. Nonetheless, one study reported a prevalence of 8%–16%, with higher rates observed in women and older individuals, mirroring patterns seen in knee OA (Kalladka et al., 2014).

Despite its significant impact on quality of life, TMJOA remains under-researched compared to knee OA. The disparity in research attention, therapeutic development, and funding likely reflects differences in perceived clinical importance. However, the high prevalence and burden of TMJOA warrant increased research efforts. Integrating insights from knee OA studies may accelerate understanding of TMJOA pathogenesis and support the development of effective treatments.

### 2.3 Symptoms and diagnosis

The European League Against Rheumatism (EULAR) defines three key symptoms for the diagnosis of knee OA: persistent knee pain, limited morning stiffness, and reduced joint function. The severity of knee OA can range from mild discomfort to joint immobilization (Heidari, 2011).

TMJOA exhibits overlapping clinical features, including joint pain, restricted mobility, and abnormal joint sounds. In early stages, TMJOA is often associated with synovitis, leading to pain, limited mouth opening, stiffness, and characteristic joint noises such as crepitus and clicking (Schiffman et al., 2014). In advanced cases, structural deformities may occur, including mandibular deviation, facial asymmetry, occlusal instability, and malocclusion (Chen et al., 2005).

Diagnostic criteria for TMJOA have been established through the Research Diagnostic Criteria for Temporomandibular Disorders (RDC/TMD), introduced in 1991 with support from the National Institute of Dental Research (NIDR) and widely adopted internationally (J. Craniomandibular Disorder Facial Oral Pain, 1992) (R and Sf, 2016). The most recent and widely accepted diagnostic framework is the Diagnostic Criteria for Temporomandibular Disorders (DC/TMD, 2014), which has replaced the older RDC/TMD system. Imaging techniques such as magnetic resonance imaging (MRI) and computed tomography (CT) are commonly employed, with MRI offering high reliability for detecting disc displacement (Schiffman et al., 2014).

For knee OA, diagnostic grading systems are more mature. The Kallgren–Lawrence system and the International Osteoarthritis Research Society criteria are widely used. In addition, advanced MRI-based scoring systems such as the Whole-Organ MRI Score (WORMS), the Boston–Leeds Osteoarthritis Knee Score, and the MRI Osteoarthritis Knee Score have further improved diagnostic precision (Guermazi et al., 2013).

In summary, while the diagnostic framework for knee OA is well-developed and standardized, TMJOA remains less established. There is a pressing need for refined scoring systems and improved imaging protocols to enhance diagnostic accuracy for TMJOA.

## 3 Pathological heterogeneity

**FIGURE 1:**
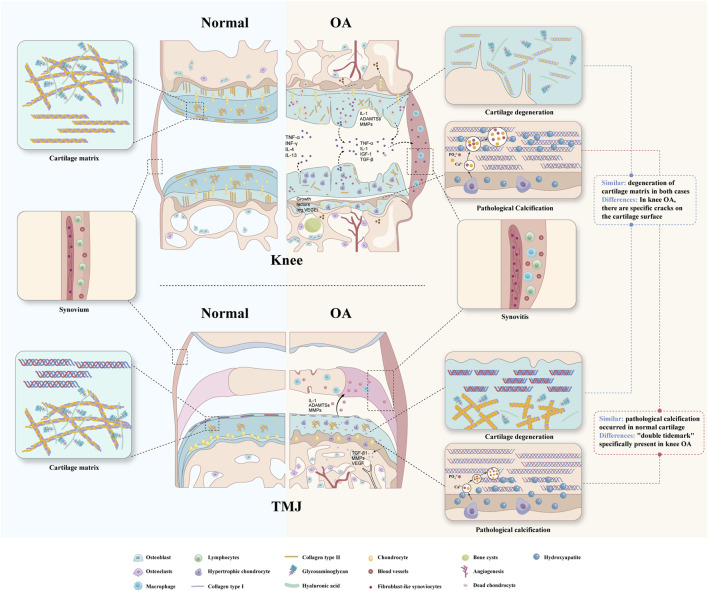
Comparison of the physiological state and pathological changes in Knee OA and TMJOA.

OA, once considered a purely mechanical disease, is now recognized as a multifactorial joint disorder involving cartilage, subchondral bone, and synovium (Figure 1). Multiple risk factors converge to disrupt tissue homeostasis, driving disease progression.

### 3.1 Cartilage

Progressive cartilage degradation is a hallmark of OA, involving extracellular matrix breakdown, proteoglycan loss, and collagen disorganization, ultimately impairing the tissue’s biomechanical function.

#### 3.1.1 Cartilage degeneration

Cartilage homeostasis in OA is disrupted by an imbalance between anabolic and catabolic processes. Excessive matrix metalloproteinases (MMPs) degrade collagen and proteoglycans. Although chondrocytes initially respond by increasing proteoglycan synthesis and secreting tissue inhibitors of MMPs (TIMPs), these compensatory efforts are insufficient. Progressive matrix loss leads to water accumulation, fiber disorganization, and structural weakening. In advanced stages, surface fissures and cracks develop, exposing the subchondral bone (Loeser et al., 2012).

In knee OA, degeneration begins with matrix depletion and progresses to fibrillation, superficial erosion, and deep-layer cracking. Fibrillation is an early indicator of hyaline cartilage damage, preceding full-thickness defects and subchondral exposure.

In contrast, TMJOA exhibits a different degeneration pattern. Surface damage is less prominent, while fissures tend to appear in deeper cartilage layers. Other features include erosive resorption, sclerosis, osteophyte formation, and subchondral pseudocysts (Wang et al., 2015). This difference may reflect the unique fibrocartilage composition of TMJ cartilage, containing both type I and type II collagen, which may confer greater reparative capacity (Wadhwa and Kapila, 2008).

These contrasting degradation patterns highlight joint-specific differences in pathology. While both conditions involve ECM breakdown and homeostasis disruption, knee OA shows more severe surface erosion due to higher mechanical loading, whereas TMJOA exhibits deeper, localized damage potentially linked to cartilage composition and loading patterns (Li et al., 2021).

#### 3.1.2 Pathological calcification in cartilage

Physiological calcification occurs in bones, teeth, and growth plates, but OA is characterized by ectopic cartilage calcification. In knee OA, calcification affects both superficial and deep cartilage layers, resulting in the characteristic “double tidemark.” Recent studies suggest dual calcification mechanisms: “top-down” mineralization at the surface and “bottom-up” from the osteochondral junction (Wang et al., 2023).

In TMJOA, calcification remains less understood. Observations include an upward shift in the calcified cartilage front and cartilage thinning, but the “double tidemark” phenomenon has not been reported (Zhang et al., 2016). Whether this reflects true mechanistic differences or research gaps remains unclear.

These disparities suggest that pathological calcification in knee OA is more advanced in both understanding and staging. Further investigation is needed to clarify whether TMJOA follows a different calcification trajectory or simply lacks comprehensive study.

### 3.2 Subchondral bone

Extensive studies on the knee have demonstrated that subchondral bone exhibits distinct pathological changes at different stages of OA. Early alterations include thinning of the subchondral bone plate, increased porosity, disruption of trabecular architecture, increased trabecular separation, and reduced trabecular thickness. These changes are followed by compensatory remodeling characterized by thickening of the subchondral plate and trabeculae, subchondral sclerosis, and reduced marrow space (Hügle and Geurts, 2016). Subchondral bone cysts and osteophyte formation observed on conventional radiographs are considered hallmarks of late-stage OA (Li et al., 2013).

In TMJOA, pathological changes of the condylar subchondral bone are considered major contributors to the radiographic manifestations of the condyle. Common degenerative changes include erosion, osteophyte,flattening, and pseudocyst-like lesions—all of which reflect subchondral bone remodeling (Cömert Kiliç et al., 2015). These subchondral alterations are frequently associated with limited mouth opening and pain, and they represent a significant etiological factor in dentofacial deformities (Li et al., 2022).

Emerging evidence indicates that subchondral bone changes in both knee OA and TMJOA share similar radiographic features, primarily driven by imbalanced bone remodeling. This imbalance—between resorption and formation—leads to reduced subchondral bone modulus (Day et al., 2001). In TMJOA, condylar degeneration is often characterized by bone loss (Demirturk et al., 2024), while knee OA studies tend to focus on altered biomechanical properties. This discrepancy likely reflects the joints’ differing functions. The TMJ endures continuous occlusal loading, and a rabbit model of TMJOA has shown that bone microarchitecture is highly responsive to the direction and magnitude of mechanical load. Even minor mechanical shifts may trigger subchondral degeneration (Fujisawa et al., 2003), highlighting the need to further investigate the biomechanical properties of the condylar subchondral bone in TMJOA.

### 3.3 Synovitis

Synovitis occurs throughout all stages of OA and impairs the joint’s lubricating function (Scanzello and Goldring, 2012). MRI studies in 39 knee OA patients revealed synovial thickening of varying severity, with fibrin deposition and inflammatory cell infiltration more pronounced in advanced stages. Synovitis, detectable even in early OA, can be assessed by MRI to classify patients in clinical trials and identify candidates for synovial-targeted therapies (Roemer et al., 2011).

Histological studies have identified the presence of intima hyperplasia, fibrosis, and increased vascularity in TMJOA, suggesting a multifactorial process that includes both immune activation and mechanical stress. This dynamic process reflects the unique biomechanical environment of the temporomandibular joint (Feng et al., 2021).The complexity of synovial micro-lesions in TMJOA suggests that immune, vascular, and mechanical factors may play distinct roles in disease progression compared to knee OA.

While synovitis is a well-established biomarker and therapeutic target in knee OA, its role in TMJOA remains underexplored. Clarifying the inflammatory mechanisms in TMJOA may reveal novel intervention points and support the development of joint-specific treatment strategies.

## 4 Pathogenic heterogeneity

The diverse pathological features observed across joint tissues in OA raise an ongoing debate: are these tissue alterations initiators of disease, or are they secondary consequences of OA progression? Relying solely on clinical manifestations and gross pathological descriptions is insufficient to resolve this question. A more nuanced understanding necessitates in-depth exploration of tissue-specific micro-lesions and their interrelated roles in OA pathogenesis.

### 4.1 Cartilage

#### 4.1.1 Chondrocyte death

Cell death is a fundamental physiological process in multicellular organisms, and recent studies have highlighted the critical role of regulated cell death in the progression of OA (Yu et al., 2021). In knee OA, various forms of chondrocyte death have been identified as key contributors to disease progression, leading to the proposal of corresponding therapeutic targets. Acid-sensing ion channel 1a (ASIC1a) and the NLRP3 inflammasome have been implicated in pyroptosis of chondrocytes in OA (Wu et al., 2019; Zhang et al., 2021). Ferroptosis has been shown to exacerbate extracellular matrix degradation (Yao et al., 2021), while autophagy dysregulation due to cellular senescence is another major mechanism under investigation (Caramés et al., 2010). A study utilizing miR-181a-5p antisense oligonucleotides (ASO) to inhibit chondrocyte apoptosis in rats and mice demonstrated that miR-181a-5p is a key pro-apoptotic factor (Nakamura et al., 2019).

Recent research in TMJOA has identified circGCN1L1, a circular RNA that promotes chondrocyte apoptosis by targeting miR-330-3p and TNF-α (Zhu et al., 2020). Additionally, signaling pathways such as Indian hedgehog (Ihh), modulated by CaMKII, drive hypertrophy by upregulating Ihh and suppressing PTH1R, whereas PTH1R signaling acts to inhibit hypertrophy through the Ihh–PTHrP feedback loop (Long et al., 2019). In TMJOA, reduced FGFR3-mediated suppression of Ihh permits the Ihh–Smo–Gli–Runx2 axis to promote hypertrophy (Yang et al., 2019). The Wnt signaling pathway is also implicated: the canonical Wnt/β-catenin pathway promotes Col X and Runx2 expression via Dnmt3b downregulation, while the non-canonical Wnt/JNK pathway induces hypertrophy and chondrocyte migration (Zhou et al., 2019).

Although cell death is recognized as a crucial driver of OA progression, its specific role in TMJOA remains poorly characterized. Most current studies in TMJOA focus on signaling pathways, with limited identification of definitive therapeutic targets.

#### 4.1.2 Matrix degeneration

Chondrocytes initiate cartilage matrix degeneration in response to excessive mechanical loading, leading to increased water content, GAGs loss, and proteoglycan degradation. Even without macroscopic joint changes, ADAMTS-4 and ADAMTS-5 cleave aggrecan core proteins from the hyaluronan backbone, disrupting GAG function and cartilage permeability (Pratta et al., 2006). Early chondrocyte compensatory synthesis precedes superficial fibrosis and matrix degradation, with MMP-13 playing a key role in type II collagen breakdown. Dysregulated anabolic and catabolic activity accelerates OA progression and structural deterioration (Loeser et al., 2012).

In knee OA, enzymatic degradation and mechanical damage primarily affect type II collagen and proteoglycans, compromising cartilage integrity. Urinary collagen type-II C-terminal cleavage neoepitope (uC2C) correlates with cartilage degradation, indicating potential as an early biomarker (Ren et al., 2023). An imbalance between collagen and proteoglycans weakens cartilage’s load-bearing capacity, exacerbating degenerative joint disease (Gauci et al., 2017).

In TMJOA, lubrication impairment alters frictional properties and surface wear, triggering pro-inflammatory mediator release and enzymatic degradation under mechanical stress. The Ras-related protein Rap-2a (RAP2A) modulates TMJOA progression via the Hippo/YAP pathway, influencing chondrocyte phenotypic shifts and matrix synthesis (Ma et al., 2020).

The primary molecular signaling pathways involved in knee OA and TMJOA differ, leading to distinct manifestations of micro-lesions. This raises a critical question: how do anatomical and biomechanical differences contribute to joint-specific pathogenesis? Investigating systemic factors such as inflammation and metabolism, along with local mechanical and molecular alterations, may be crucial for a deeper understanding of OA heterogeneity.

#### 4.1.3 Pathological calcification

Basic calcium phosphate (BCP) and calcium pyrophosphate dehydrate (CPPD) are key pathological minerals in joint diseases (Rosenthal, 2018). CPPD crystals, linked to acute inflammatory arthritis and degenerative conditions, deposit in joints, spine, and soft tissues, causing chondrocalcinosis and worsening OA symptoms.

Extensive studies on knee OA have demonstrated that initial calcification presents as nanosphere formation, progressing to the erosion of deeper, more compact structures in advanced stages, corresponding to chondrocyte apoptosis and hypertrophy. These chondrocytes contribute to mineralization, influenced by the balance between inhibitors (proteoglycans, collagen-II) and promoters (collagen-I, collagen-X, Runx-2), which degrade the matrix and favor calcium phosphate deposition. This calcification disrupts cartilage ECM, alters mechanical properties, and accelerates OA progression (Hu et al., 2021).

Collagen fiber fragmentation and disorganization, fiber gap enlargement, matrix vesicle generation and increased mineral deposition in the matrix surrounding hypertrophic chondrocytes in cartilage were observed after abnormal stress-related surgery on the rat TMJ and intensified over time (Zhang et al., 2016). Pathological calcification follows a “bottom-up” pattern and is accelerated by chondrocyte-derived exosomes. Lysyl oxidase (LOX), a key enzyme in collagen cross-linking, promotes cartilage calcification, and its inhibition has potential to reduce mineralization (Wang X. et al., 2022).

Pathological calcification is a complex organic-inorganic interaction. Current research on pathological calcification primarily focuses on the cardiovascular system. The receptor activator of nuclear factor κB (RANK)/RANK ligand (RANKL)/osteoprotegerin (OPG) system plays a fundamental role in bone metabolism. Numerous studies have demonstrated that increased RANKL levels and decreased OPG levels are associated with the promotion of vascular calcification (Fernández-Villabrille et al., 2024). However, the specific mechanisms by which the RANK/OPG axis contributes to pathological calcification in OA remain largely unexplored. Future studies on OA may benefit from insights gained in cardiovascular research, particularly in elucidating the mechanisms of pathological calcification across different joints and developing strategies to prevent or mitigate its progression.

### 4.2 Subchondral bone

The micro-lesion remodeling process of subchondral bone appears largely conserved across different joints affected by OA. It is characterized by increased osteoclast-mediated bone resorption and dysregulated osteoblast-mediated bone formation. Aberrant mechanical strain disrupts osteoblast homeostasis, marked by elevated expression of interleukin-6 (IL-6), prostaglandin E2 (PGE2), matrix metalloproteinases (MMPs), and receptor activator of nuclear factor κB ligand (RANKL), alongside reduced osteoprotegerin (OPG) production (Ni et al., 2011). In early OA, apoptotic osteocytes upregulate RANKL to activate osteoclasts and promote bone resorption. As OA progresses, ongoing cartilage degradation increases mechanical loading on subchondral bone, prompting osteocytes to adapt by upregulating Wnt signaling and suppressing sclerostin (SOST) expression (Li et al., 2019). Additionally, studies analyzing tibial plateau samples from OA patients have shown that transforming growth factor-β1 (TGF-β1) in osteocytes enhances osteoblast-driven bone anabolism in late-stage OA via activation of Smad2/3 signaling (Dai et al., 2020).

Key cytokines such as RANKL and TGF-β1, which mediate subchondral bone remodeling in knee OA, appear to play similar roles in TMJOA (Corrado et al., 2013). Notably, estrogen and progesterone may directly influence TMJOA progression by acting on bone cells. Estrogen suppresses osteoclast activity through the Wnt pathway, while progesterone inhibits bone resorption via NF-κB signaling. (Xue et al., 2017; Ye et al., 2018). Although current evidence is limited, these mechanisms align with the markedly higher prevalence of TMJOA in females. In contrast, knee OA shows less pronounced sex-related differences, suggesting the existence of TMJ-specific targets responsive to estrogen and progesterone that warrant further investigation.

### 4.3 Synovitis

Synovial micro-lesions in knee OA marked by mononuclear cell infiltration and lining cell proliferation, in many early or mild OA patients (de Lange-Brokaar et al., 2014).

In TMJOA, synovial micro-lesions exhibit various histological changes. Muto observed synovial hyperplasia, cell loss, and fibrin deposition following trauma. Nozawa-Inoue reported enhanced vascularization and synovial lining thickening in an arthritis model, suggesting immune involvement (Muto et al., 2003). Dijk Graaf identified intima hyperplasia and fibrosis in the synovial membrane during different stages of TMJOA (Dijkgraaf et al., 1997).

Synovitis occurs throughout all stages of OA and impairs joint lubrication. In knee OA, NF-κB activation and NLRP3 inflammasome pathways are key mediators of synovial inflammation, leading to cytokine release and cartilage damage (Goldring et al., 2011). In TMJOA, NF-κB signaling has also been implicated, but additional factors such as HMGB1-induced angiogenesis, enhanced vascularization, and the influence of mechanical loading and sex hormones play distinct roles. While both conditions involve inflammatory activation, TMJOA appears to exhibit more pronounced vascular and hormone-related responses (Ou et al., 2021).

While synovitis appears early and persists in OA, its precise triggers and progression mechanisms remain unclear, particularly regarding systemic *versus* localized factors. The histological variability in TMJOA suggests distinct immune, vascular, and mechanical contributions across joints. Future research should explore early synovial changes as potential biomarkers or therapeutic targets, enabling joint-specific disease modulation.

## 5 Discussion

Despite significant progress in OA research, major challenges persist in elucidating the divergent pathophysiological mechanisms and developing optimal treatment strategies for both knee OA and temporomandibular joint osteoarthritis TMJOA. While innovations in imaging techniques and biochemical markers have enhanced diagnostic accuracy and therapeutic monitoring, fundamental knowledge gaps remain—particularly regarding the joint-specific biological processes that underlie disease initiation and progression.

The anatomical and biomechanical differences between the knee and the TMJ) are central to their distinct disease trajectories. Knee OA primarily results from chronic axial loading and repetitive weight-bearing stress, leading to superficial cartilage erosion and well-characterized subchondral remodeling. In contrast, TMJOA is driven by multidirectional masticatory forces, neuromuscular feedback, and craniofacial biomechanics, contributing to unique pathological features such as deep-layer cartilage fissures, fibrocartilage-specific degeneration, and distinct calcification patterns. These disparities highlight the need for joint-specific mechanistic research to improve pathophysiological understanding and therapeutic targeting.

Decades of research have positioned knee OA as a well-characterized model of joint degeneration, supported by validated preclinical models, extensive molecular mapping, and established clinical protocols. TMJOA, by contrast, remains comparatively underexplored. The lack of standardized diagnostic criteria validated imaging-based grading systems, and large-scale clinical trials continues to limit progress in the field. As emphasized in this review, TMJOA research is further constrained by a scarcity of robust animal models and a disproportionate gap in basic and translational studies compared to knee OA. Addressing this imbalance requires concerted, interdisciplinary efforts involving oral and maxillofacial specialists, rheumatologists, musculoskeletal radiologists, and biomedical researchers.

Knee OA and TMJOA share key pathological features—cartilage degeneration, subchondral bone remodeling, and synovitis—but differ in histological composition, mechanical environment, and calcification patterns. Knee OA is characterized by surface cartilage fissures, double tidemark calcification, and well-established diagnostic frameworks, whereas TMJOA presents with deep-layer fissures, absent double tidemark, and less standardized diagnostic criteria. These similarities and differences underscore the importance of cross-joint comparisons to identify both shared mechanisms and joint-specific targets for future therapies.

Emerging regenerative therapies—including exosome-based treatments, mesenchymal stem cell applications, and cartilage tissue engineering—show considerable promise in restoring joint function and delaying disease progression. However, their clinical translation remains hindered by heterogeneity in patient responses, insufficient long-term outcome data, and regulatory limitations. Although such strategies have been extensively investigated in knee OA, their application in TMJOA remains in its infancy. Dedicated exploration of TMJOA-specific regenerative approaches may not only accelerate clinical advances in this neglected joint but also offer transferable insights into tissue-specific therapeutic design across the OA spectrum.

Looking ahead, future research should prioritize the integration of molecular, biomechanical, and clinical datasets to uncover both shared drivers of OA and joint-specific mechanisms. Precision medicine approaches—such as omics-based profiling, patient stratification, and computational modeling—hold great potential for developing personalized intervention strategies. For example, the semiquantitative MRI scoring system established for knee OA, which evaluates joint cartilage, meniscus, osteophytes, bone marrow abnormalities, synovitis, and effusion, may serve as a valuable reference for improving the diagnostic framework of TMJOA.

By systematically addressing the unique anatomical, functional, and molecular characteristics of TMJOA, while leveraging the extensive knowledge base of knee OA, the field may advance toward a more comprehensive, mechanism-driven, and individualized framework for OA management.
